# Supplementary material for: Emergent symmetries in block copolymer epitaxy
Source: Nat Commun. 2019 Jul 5;10:2974. doi: 10.1038/s41467-019-10896-5 (PMC6611865; doi:10.1038/s41467-019-10896-5)
Supplement: Supplementary file 2 — Supplementary Information [file 41467_2019_10896_MOESM2_ESM.pdf]

Supplementary Information for:

Emergent Symmetries in Block Copolymer Epitaxy

Ding et al.

Supplementary Methods

SiO_x posts were generated through electron-beam lithography (EBL) of hydrogen silsesquioxane (HSQ) and subsequent etching (S*1*). In a typical experiment, 4% HSQ was spincoated on to silicon substrate to a thickness of 45nm. The sample was then exposed by EBL (Elionix ELS-F125) with 125kV acceleration voltage and 1nA current. Salty developer, *i.e.*, 4wt% NaOH and 1wt% NaCl was used to develop the exposed HSQ sample. The sample was then rinsed by de-ionized (DI) water for 2 minutes and isopropanol for 10 seconds before drying by N_2.

The posts were grafted with homopolymer hydroxyl-terminated polystyrene (PS-OH, 7.0kg mol^{-1}, from Polymer Source Inc.) and the as-formed PS brush layer was used to attract the PS block in the BCP. We used a PS-*b*-P4VP block copolymer which formed a perforated lamellar phase in thin films under thermal annealing conditions (24.0kg mol^{-1} for PS block and 9.5kg mol^{-1} for P4VP block, f_{P4VP} = 30%, PDI = 1.15, from Polymer Source Inc.); it is noteworthy that the P4VP block formed the mesh skeleton under thermal annealing, with equilibrium inter-domain periodicity L_0 = 43$\pm$1nm in the un-templated sample). A 37nm thin film of this BCP was deposited through spin-coating. The thin film underwent 48 hours of thermal annealing at 200°C in vacuum oven (20torr). Then, the sample was immersed in inorganic salt/acid mixed solution (e.g., H_2PtCl_6 aqueous solution, 20mmol L^{-1}, with 0.9mol HCl) for 1 minute to 30 minutes. The function of this step is two-fold: 1) to protonate P4VP block and turn it into a positively charged polyelectrolyte chain; 2) to incorporate negatively-charged inorganic salt into P4VP block. Further, oxygen plasma etching (1 minute to 5 minutes) was performed to remove the carbon-based BCP backbones and to transform inorganic salts into metal or metal oxide. The posts were subsequently removed by immersing the sample into an HF solution for 30 seconds. SEM images were taken by using a Zeiss Merlin high resolution SEM with an acceleration voltage of 10kV.

Different conditions of film thicknesses (from 35nm to 65nm) and thermal annealing temperatures (from 180°C to 230°C) were also tested for both untemplated and templated PS-*b*-P4VP thin film. In all cases, only perforated lamellae phase was observed. When the average film thickness reaches above 50nm, double layered perforated lamellae phase thin film started to emerge. Therefore, in order to observe the broken symmetry phenomenon, the film thickness of the templated thin film should be controlled under 50nm.

Supplementary Figures

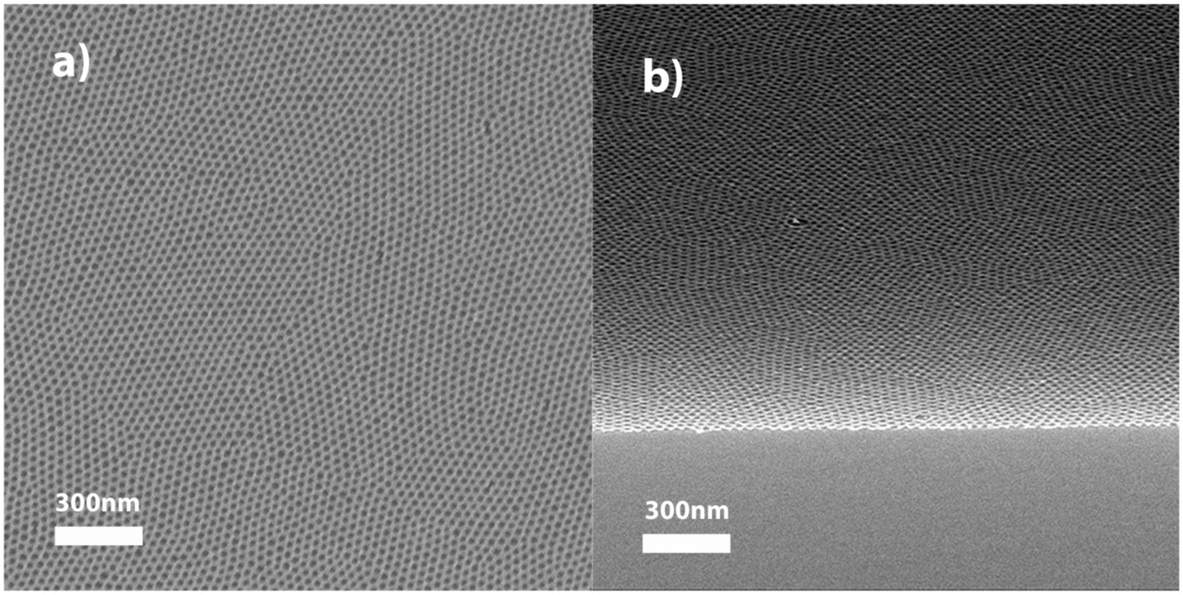

Supplementary Figure 1. SEM characterization of un-templated Platinum mesh structure derived from thermally annealed PS-*b*-P4VP BCP: a) top-down view; b) viewed at 70° angle, with the edge of the substrate showing at the bottom of the image.

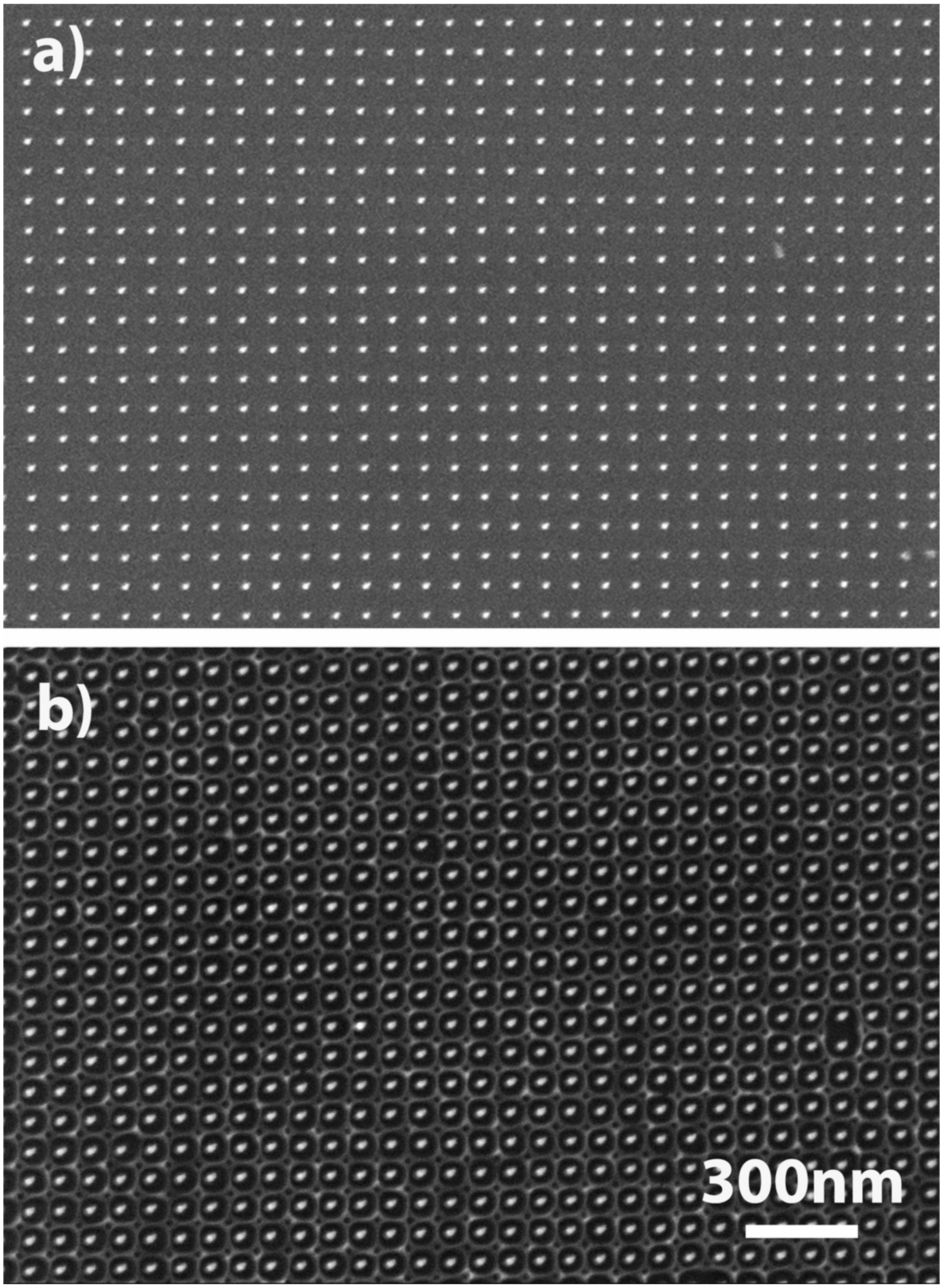

Supplementary Figure 2. SEM characterization of (a) HSQ post array templates and (b) BCP in template (top-down view, after metallization and etching).

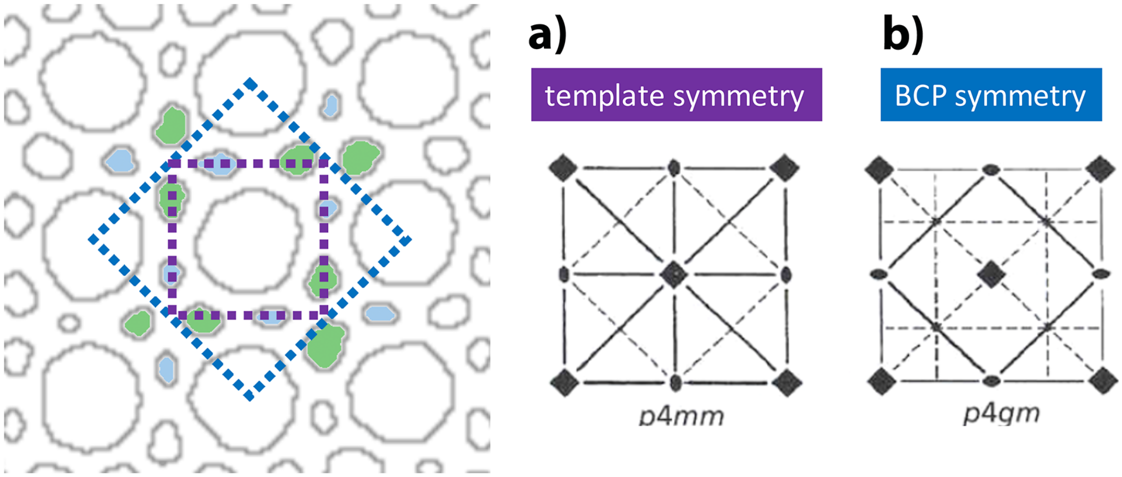

Supplementary Figure 3. Symmetric operations in a) ***p4mm*** and b) ***p4gm*** plane symmetry groups.

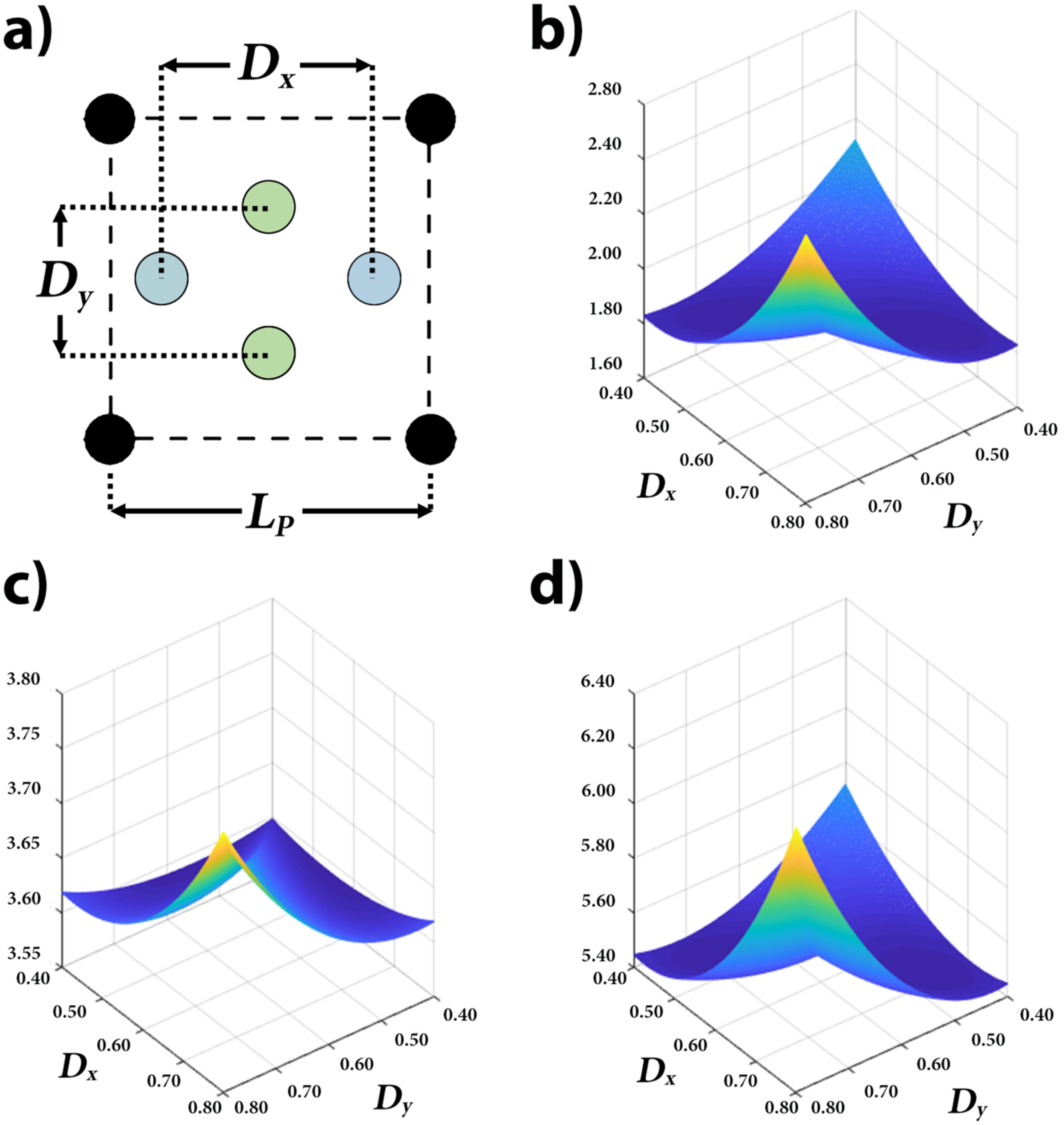

Supplementary Figure 4. a) Schematics of parameters D_x and D_y in the analytical model. Free energy landscape with respect to different D_x and D_y's (in unit of L_p): b) entropic stretching energy, with minimum at $D_x = 0.48$ and $D_y = 0.68$; c) enthalpic interface energy, with minimum at $D_x = 0.40$ and $D_y = 0.55$; d) total free energy with minimum at $D_x = 0.48$ and $D_y = 0.68$.

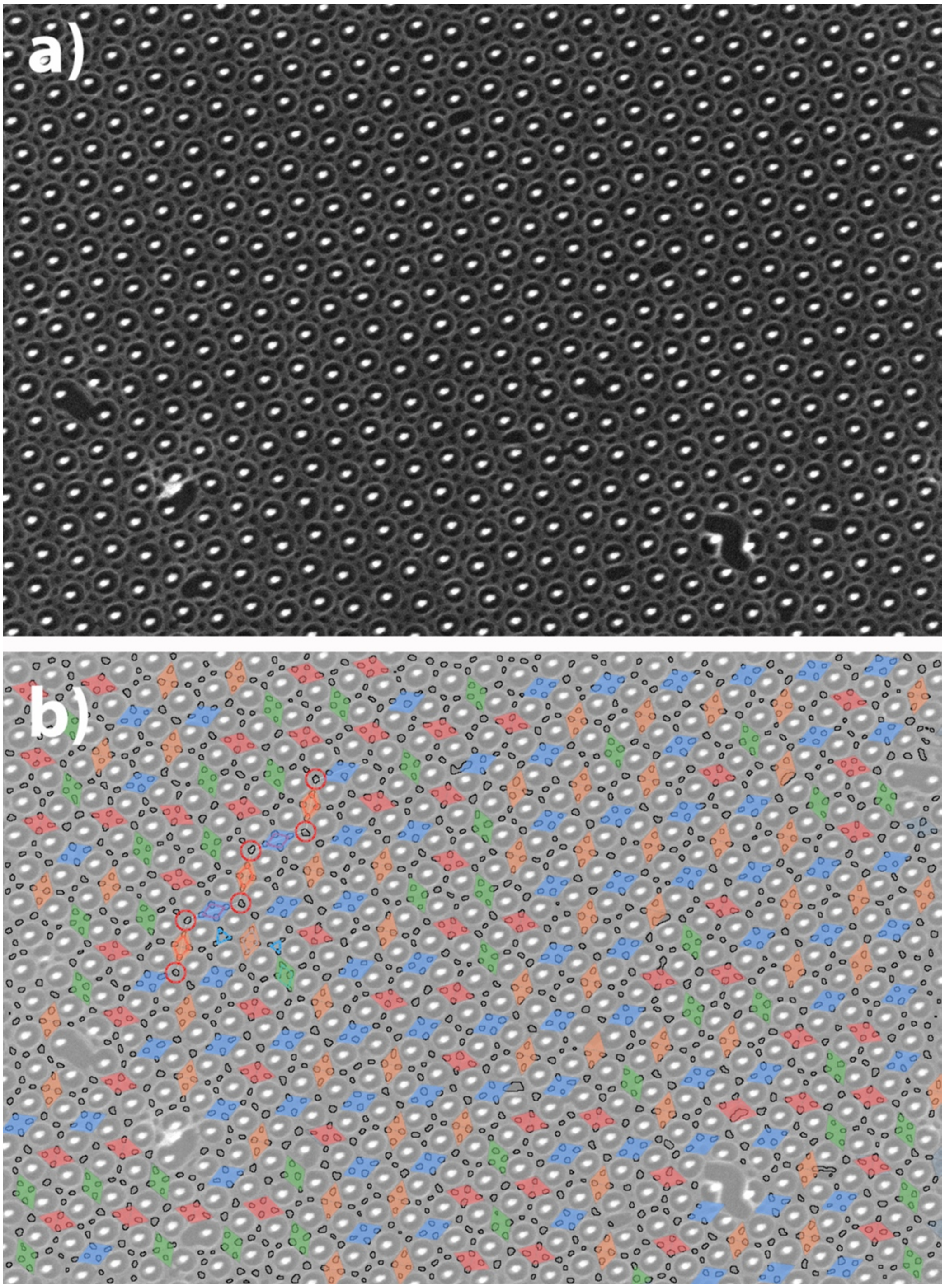

Supplementary Figure 5. a) SEM of BCP in 3^2434 Archimedean tiling template. b) different orientations of the rhombuses labeled by 4 different colors. Note that orange and blue constitute a phase (the blue domains in Fig. 3a) while red and green indicate the degenerate phase of the other orientation (the green domains in Fig. 3a).

Supplementary Discussion

SCFT Simulations. SCFT was employed to simulate post templated DSA of BCP. Details of the formalism can be found in a previous study (S*2*). For the purpose of this work, we chose $f = 0.7$ where f is the volume fraction of polymer A, making A the majority block. Different values of χN ($15 < \chi N < 23$) and different thickness of brush layers were used with no direct effect on the BCP self-assembled pattern (Fig. S6).

The results of square grid post template were implemented using $\chi N = 15$ and the 3^2434 post pattern using $\chi N = 17$. Here, N refers to the degree of polymerization and χ is the Flory-Huggins parameter. Both blocks have equal statistical segment length. Simulations are conducted in 2D assuming no variation taking place across film thickness. The computational grid has a pixel size of $0.12R_g$ where R_g is the polymer radius of gyration. The overall size (height and depth) of the computational cell was modified by changing the total number of pixels.

A masking method was employed to mimic the effect of the post pattern in directed self-assembly. A pressure field $w_+ = (w_B + w_A)/2$ was imposed as a mask where posts are located to create polymer inaccessible areas. A magnitude of $w_+ = 8$ was applied to a circular post of diameter 16 pixels. Pattern chemical affinity towards block B was achieved using an exchange potential $w_- = (w_B - w_A)/2 = -8$, surrounding the posts with a thickness of 10 pixels.

For the initial study of 3^2434 template, we used squares and triangles of equal side length. This fixed the angle of the rhombus to 60°. The simulation results reproduced the essential phases observed experimentally: 2,3,5 domains inside a rhombus in the range of L_p = 2.10 to 2.50 L_0 (denoted as II, III, and V in Fig. S7a, S7b, and S7d respectively). Nonetheless, defective mixed phases were frequently observed with a probability that depends on the computational cell size. Hence, additional computational variables were explored, namely the rhombus angle θ (Fig. 3d and Fig S7). Fig. S7h shows the free energy landscape as a function of L_p and θ. It is evident that in the range studied in this work, the landscape is dominated by three plain phases of 2, 4, and 5 (Fig. S7a, S7c, and S7d, respectively).

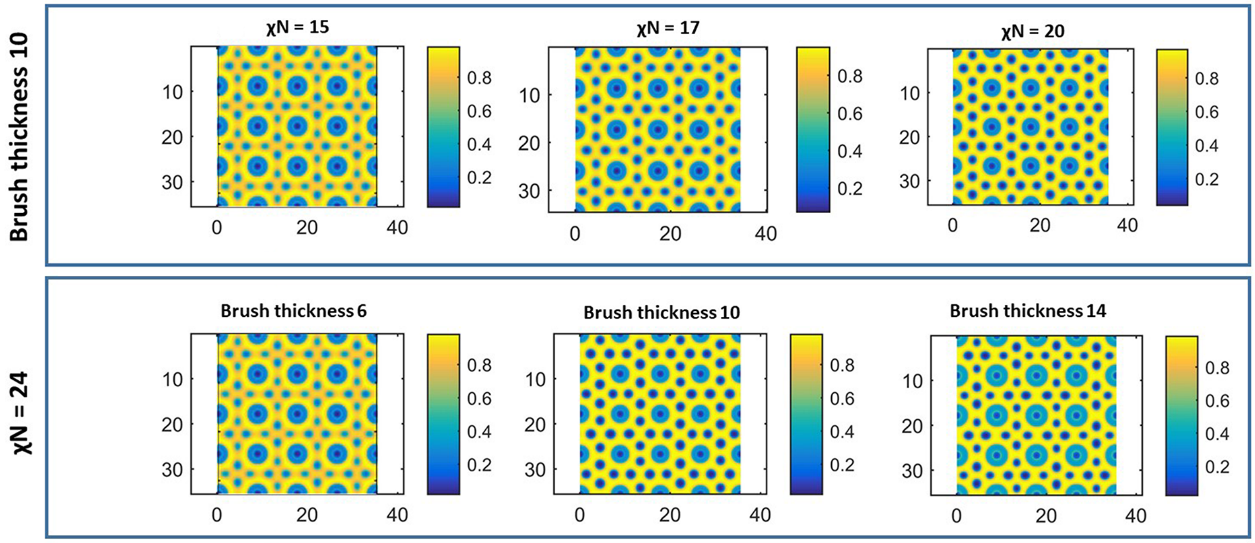

Supplementary Figure 6. Alternating rhombus structures formed by PS domains with *p4gm* symmetry in template at different χN's and brush layer thicknesses. Top row: different χN's with fixed brush layer thickness (at 10); bottom row: different brush layer thickness with fixed χN (at 24).

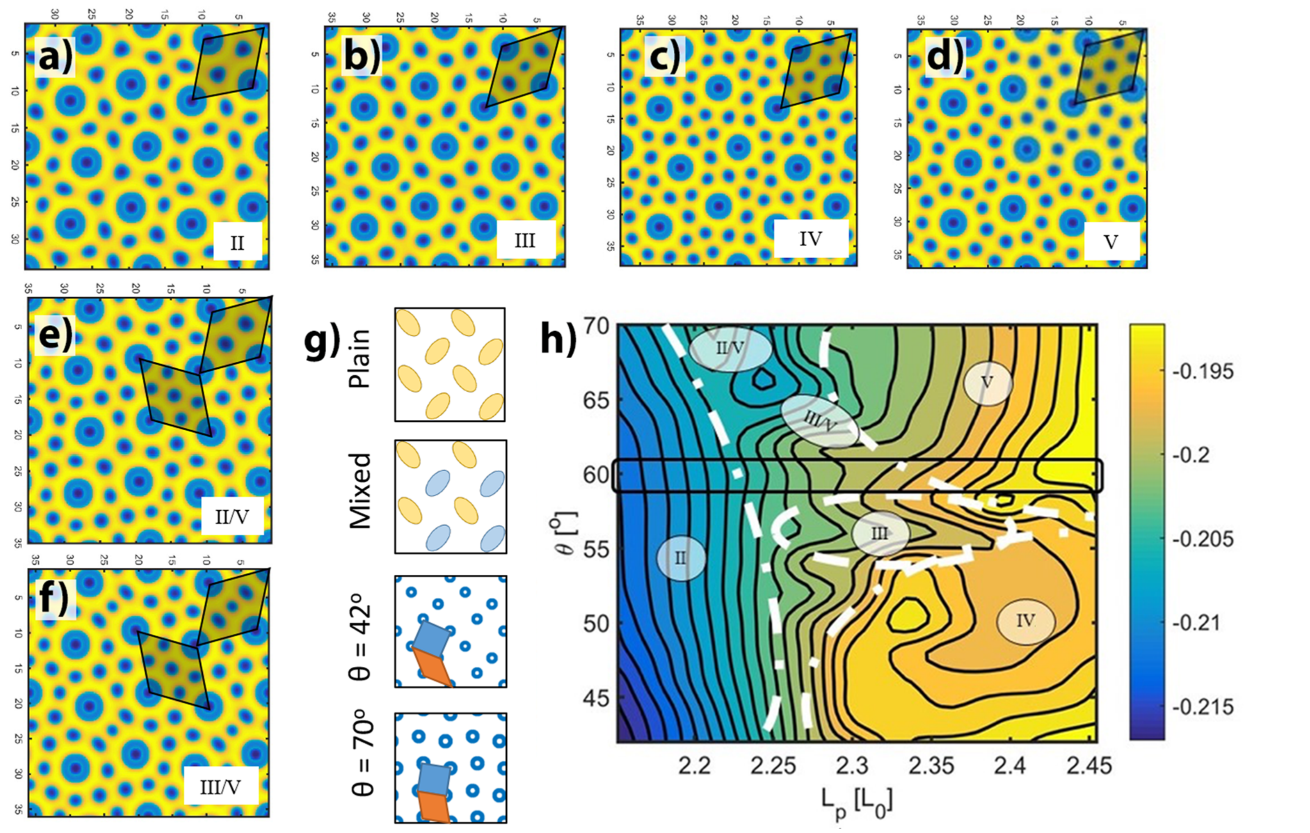

Supplementary Figure 7. a - f) different arrangements of PS domains 3^2434 template, with highlights of the rhombus area enclosed by the templates. The types are indicated on the bottom right corner of each graph. g) Illustrations of mixed type vs plain type, and different angles for the templating rhombuses. h) energy landscape of BCP in 3^2434 template.

Qualitatively, phase II effectively occupies the small $L_p < 2.25\ L_0$ independent of θ. Large $L_p > 2.3\ L_0$ shows phase V for $\theta > 60^o$ while phase VI is located where $\theta < 60^o$. Plain phase III is confined to a small stability window of $2.3L_0 < L_p < 2.4L_0$ and θ of 54°. More interestingly, stable mixed phases II/V and III/V were observed for $\theta > 64^o$ and $L_p \sim 2.3L_0$ as shown in Fig S7e and S7f. The mixed phases consisted of rows with an alternating number of domains inside the rhombus template. Indeed, the stable mixed phases show lower symmetry with a supercell size of twice that of the post. Clear BCP rhombus configuration is observed inside the square arrangement of the 3^2434 template for mixed phases. The orientation of the rhombus is an indication of the communication across template regions as the short axis of the rhombus points towards the 5 phase, while the long axis points towards the 2 or 3 phases. The unequal number of BCP domains inside the rhombus template causes the four BCP domains inside the square template to deform into a rhombus shape. Conversely, a square configuration prevailed inside the square template due to equal tension of the surrounding domains for plain phases. The free energy landscape could explain the observed experimental results. For $\theta = 60^o$ and varying L_p, the polymer configurations are located in the vicinity of multiple plain phases and far from mixed phases. Hence, we see the ubiquitous presence of plain phase signatures with no long-range order.

Strong-stretching theory (SST) calculations. In this work, we employ the strong-stretching theory (SST) calculations by Milner and Olmsted (*S3, S4*) for circular domains in the polyhedral interface limit (PIL). Here, a simple assumption for chain distribution (the straight-path ansatz) is employed, where the AB interface adopts the same shape as the lattice Voronoi cell.

For a linear AB diblock, the total segment number in a chain $N = N_A + N_B$, where the fraction of A is $f = N_A/N$. Segments of both blocks have equal volume and equal statistical segment length. The total free energy $F\ [nk_bT]$ consists of F_i representing the interfacial energy between blocks at the core/corona interface, and F_{st} representing the entropic cost of stretching of a Gaussian chain.

(1) $F = F_i + F_{st}$

(2) $\dfrac{F_i}{nk_bT} = \dfrac{\sqrt{\chi N}}{V} A_i R_g$

Where A_i is the total interfacial area for the core/corona contact and V is the total system volume. The entropic component of free energy is calculated using SST expression derived from the "parabolic brush" assumption

(3) $\dfrac{F_{st}}{nk_bT} = \dfrac{3\pi^2}{48 R_g^{\,2} V}\left(\dfrac{1}{f^2}\int_{V_A} d^3r z^2 + \dfrac{1}{(1-f)^2}\int_{V_B} d^3r z^2\right)$

Here the integral is taken over the entire block volume. Coordinate z is a radial distance from the AB interface, where junction points are localized. To map $F[n_kbT]$ as a function of domain arrangement inside the square template, the BCP micelles are assumed to be polyhedral unit cells that cover the entire space. These unit cells are the Voronoi cells of the lattice. For an asymmetric configuration (rhombus configuration), the BCP domains occupy unequal space where hexagonal and pentagonal unit cells are constructed. In the symmetric case, all BCP domains occupy pentagonal unit cells (Fig. 2e). In this regard, for every point in the *F* plot (Fig. 2a), the BCP domains are positioned and the space is divided into the corresponding Voronoi cells. The corresponding integrals were numerically calculated.

Supplementary References

S1. I. Bita *et al.*, Graphoepitaxy of self-assembled block copolymers on two-dimensional periodic patterned templates. *Science* **321**, 939-943 (2008).

S2. K. R. Gadelrab *et al.*, Limits of Directed Self-Assembly in Block Copolymers. *Nano Lett* **18**, 3766-3772 (2018).

S3. P. D. Olmsted, S. T. Milner, Strong-Segregation Theory of Bicontinuous Phases in Block-Copolymers. *Phys Rev Lett* **72**, 936-939 (1994).

S4. P. D. Olmsted, S. T. Milner, Strong segregation theory of bicontinuous phases in block copolymers. *Macromolecules* **31**, 4011-4022 (1998).
